# The good, the bad, and the ugly: opportunities, challenges, and pitfalls in spatial proteomics modeling

**DOI:** 10.1093/bib/bbag169

**Published:** 2026-04-15

**Authors:** Shahil Yasar Haque, Swakkhar Shatabda, Salekul Islam, Shoaib Ahmed Dipu, Riasat Azim

**Affiliations:** Department of Computer Science and Engineering, United International University, United City, Madani Avenue, Badda, Dhaka 1212, Bangladesh; Department of Computer Science and Engineering , BRAC University, Kha 224, Pragati Sarani, Merul Badda, Dhaka 1212, Bangladesh; Department of Electrical and Computer Engineering, North South University, Block B, Bashundhara Residential Area, Dhaka 1229, Bangladesh; Department of Computer Science and Engineering, BRAC University, Kha 224, Pragati Sarani, Merul Badda, Dhaka 1212, Bangladesh; Department of Computer Science and Engineering, United International University, United City, Madani Avenue, Badda, Dhaka 1212, Bangladesh

**Keywords:** spatial proteomics, cellular heterogeneity, protein localization, multi-omics

## Abstract

Spatial proteomics has become one of the mainstays of spatial biology at an impressive speed. It enables the in-depth study of protein abundance, localization, and microenvironmental context at the levels of tissues, cells, and subcellular structures. Preserving the native tissue architecture and capturing the functional molecular states that cannot be deduced from transcriptomics, spatial proteomics is breaking new grounds in cellular heterogeneity, small-unit physiological niches, and disease-related tissue organization. This review covers the major strengths (”Good”) of the field, including mass spectrometry imaging, single-cell, and subcellular proteomics, by which these technological advancements in protein biology mapping at different scales have been merged. This review also discussed the limitations and trade-offs (”Bad”) that consist of issues that cause throughput bottlenecks, sensitivity constraints, antibody specificity problems, and multi-omics integration challenges. Afterwards, this review also points out the pitfalls (”Ugly”) that can result in misguided research judgments associated with false spatial gradients, protein delocalization, segmentation artifacts, batch effects, and improperly validated spatial maps. Finally, the review discusses the future research directions that can lead spatial proteomics towards greater advancement, which will lead to revolutionary improvements in biological mechanisms, translational research, and precision medicine.

## Introduction

Biological systems are inherently spatial. Organized tissues are composed of cells and subcellular components, which provide a discrete microenvironment, and protein functions are often dependent on precise localization. Classical proteomics, which typically implies the homogenization of tissue samples or the lysis of cells, destroys this localization, and spreads protein datasets across populations, obscuring important biological diversity and context. Spatial proteomics substitutes that dimension with the depths of molecules, and localizing those positions, which is an effective view into the processes and spatial locations of proteins in their natural environment [1].

Spatial proteomics is an emerging discipline leveraging recent advances in mass spectrometry (MS), imaging, and computational modeling to map protein distributions in tissues and cells [2]. Unlike spatial transcriptomics, spatial proteomics directly measures the functional molecules that execute cellular programs, capturing post-translational modifications and protein localization that cannot be inferred from mRNA alone [3].

The field of spatial proteomics is especially emerging to be useful in the direction of clinical and translational research. As an example, the spatial proteomic maps have identified patient-specific protein signatures, immune cell-stromal interactions, and tumor microenvironment niches [4]. Potential applications for biomarker discovery, precision medicine, and patient stratification can be found in these findings.

Spatial proteomics has a lot of potential, but it also has a lot of technical and computational obstacles to overcome. These include low sample input, batch effects, data integration, and reproducibility. Which is why more sensitive MS instruments, advanced computational tools and automated workflows will be needed to further progress the potentials of this field.

Recent reviews take complementary angles when discussing about spatial proteomics, but gaps associated with the limitations and error sources are not clearly reported. Papers focused on diseases primarily showcase the biological and translational capabilities of spatial proteomics in cancer, neurology, and cardiovascular research, outlining the challenges and future directions [2, 5]. While other reviews discuss the methodological landscape, differentiating targeted antibody-based imaging techniques and untargeted MS techniques; presenting their multiscale profiling capabilities [4]. Whereas others survey the extent of biochemical and subcellular localization methods [6]. Other papers highlight the computational and data analytics processes, detailing software limitations in single-cell and spatial proteomics [7]. All of these reviews provide an essential outlook into spatial proteomics, but are unable to provide detailed critiques into experimental artifacts, analytical failures, and the risks of misinterpretation in spatial proteomics as a whole.

**Fig. 1.**
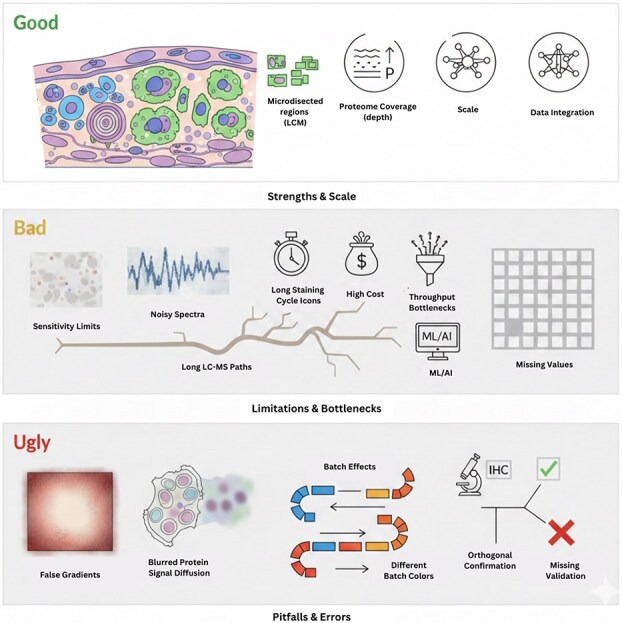
This diagram summarizes the current state of spatial proteomics by detailing its strengths, limitations, and critical pitfalls. Good: The top panel highlights the core advantages, including the ability to analyze proteins within microdissected regions of heterogeneous tissue, achieving high proteome coverage, and enabling data integration. Bad: The middle panel addresses the challenges and limitations, including sensitivity constraints, noisy spectra, high cost, throughput bottlenecks and missing data, resulting in often requiring ML/AI for corrections. Ugly: The bottom panel warns against critical data quality issues and analytical errors. These pitfalls include spatial artifacts like false gradients, blurred protein signal diffusion, significant batch effects across different runs, the frequent lack of orthogonal confirmation, and missing validation, all of which can compromise the reliability of findings.

This review is organized to discuss not only the strengths of spatial proteomics, but also its limitations and under-addressed gaps that can negatively influence biological conclusions. This review is structured to categorize and analyze sources of errors, throughput trade-offs, and sample-preparation artifacts, and also computational failures such as segmentation errors, batch effects, and false spatial gradients. Previous review do acknowledge validation as important [5, 7], but they do not illustrate how technical noise and preprocessing can easily camouflage themselves as biological signals. By showcasing the risks and problems associated with misinterpretation, reproducibility, downstream bioinformatics modeling, and integration; this review attempts to provide a caution aware guide for future research and studies to be conducted. Figure 1 illustrates the “Good,” “Bad,” and “Ugly” of spatial proteomics as discussed in this review. Our goal is to help determine the current state of spatial proteomics and its future potential.

## The good

Unlike traditional proteomics, spatial proteomics identifies the abundance and spatial context of a protein at its location in a cell, tissue, or organ. These spatial information are essential, as different cell types, microenvironments, post-translational modifications (PTMs), and subcellular localization all affect biological activities [2, 4]. Spatial proteomics can also preserve the natural structure of tissues and provide insight into cell-type diversity, microenvironmental influences, and region-specific molecular changes, all of which would have been lost in bulk assays. Spatial proteomics also helps to better visualize and understand tissue biology, cell interactions, and disease mechanisms, as it is able to integrate with numerous methods and data types [8]. This is because the localization data of proteins can be correlated with histology, transcriptomics, metabolomics, and many other data modalities.

### Overall strengths of spatial proteomics

The most important aspect of spatial proteomics is that it can associate the quantity of the protein with its location, which is very important because proteins do not act uniformly throughout a tissue. Through the application of spatial proteomics, it becomes possible for scientists to see the local signaling events, the phenotypes that are particular to the cellular neighborhood, and the patterns of expression that are dependent on the region, which in turn affect development, immune response, and disease progression [9]. Because there is often a great difference between the expression of proteins and their corresponding RNA levels, the use of spatial proteomics can provide more accurate functional information than that obtained with transcriptomics alone [10].

Moreover, by using spatial proteomics, PTMs, protein complexes, proteoforms, and enzyme activities can be separated, revealing the biochemical regulation that otherwise could not be understood from gene expression. On the other hand, the modern techniques are able to operate at different spatial scales ranging from whole tissue sections to subcellular nanodomains, thereby providing research activities with a flexible toolkit that is suitable for a wide variety of biological questions [11, 12].

The rapidly evolving computational ecosystem is another important benefit. The application of machine learning (ML), image analysis, graph modeling, and spatial statistics is becoming increasingly important in protein detection, segmentation, clustering, and multimodal data integration. So the more complex and higher the resolution the datasets become, the more computational techniques will be able to apply to spatial proteomics. In general, spatial proteomics provides a one-of-a-kind, detailed, functional, and spatially mapped picture of protein biology that is not only complementary to, but also augments, the view offered by other spatial omics approaches [6].

### Major spatial proteomics techniques

**Fig. 2.**
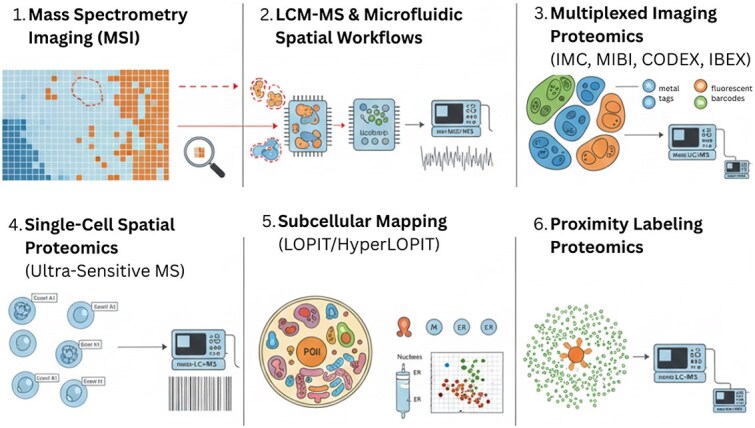
A conceptual overview of the major spatial proteomics technologies. From left to right: (1) MSI visualizes protein/peptide distributions directly in intact tissue. Untargeted protein/peptide imaging; $\mu $m–100 $\mu $m pixel resolution; maps molecular distributions across intact tissue. (2) LCM-MS and microfluidic workflows enable deep proteome profiling of microregions. High-resolution sampling; deep proteome coverage; ideal for rare tissue region and defined anatomical structures. (3) Multiplexed imaging proteomics provides high-plex single-cell spatial phenotyping. 30–60+ protein markers; single-cell resolution; maps cell phenotypes and spatial neighborhoods. (4) Single-cell spatial proteomics links ultrasensitive MS to spatially indexed cells. Single-cell proteome depth; maps cellular heterogeneity with preserved location. (5) LOPIT maps thousands of proteins to subcellular compartments. Quantitative mapping of organelle proteomes; resolves subcellular localization across thousands of proteins. (6) Proximity labeling identifies nanoscale protein neighborhoods and interaction partners. Nanoscale mapping of interaction neighborhoods; captures transient and compartment-specific interactomes *in vivo*.

This subsection, discusses the strengths of major spatial proteomics techniques. Figure 2 illustrates these techniques.

#### Mass spectrometry imaging

**Fig. 3.**
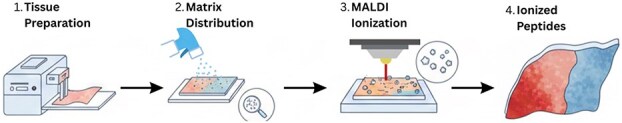
Workflow of MSI for spatial proteomics. (1) Tissue is cryo-sectioned and mounted onto a conductive glass slide for MSI. (2) Matrix is uniformly deposited *via* spraying or sublimation, embedding peptides for ionization. (3) A UV laser raster-scans the tissue pixel by pixel, causing matrix-assisted ionization of peptides. (4) Ionized peptides are extracted into the mass spectrometer, separated by mass-to-charge ratios (*m*/*z*).

Mass spectrometry imaging (MSI) is one of the leading and most promising techniques used in spatial proteomics, enabling a non-invasive and neutral procedure for mapping the mixture of peptides, proteins, lipids, metabolites, and the like and discovering their whereabouts in the tissue sections. Figure 3 illustrates the MSI process.

MSI is a method that offers a powerful, label-free, and multiplexed molecular detection approach. Since MSI does not involve the use of antibodies, fluorescent labels, or other molecular tags, it keeps the original chemical composition of the tissue intact. In a single MSI analysis, one can identify hundreds to thousands of molecular species, metabolites, lipids, peptides, and even proteins, dispersed across a tissue section, thus creating an extensive and impartial molecular picture of the sample [13].

MSI allows the preservation of tissue architecture and spatial context, using thin fresh-frozen or Formalin-Fixed Paraffin-Embedded (FFPE) tissue segments while maintaining histology and morphology. This provides a direct correlation of molecular distributions with tissue structures, tissue regions, and disease features directly [14, 15]. Secondly, the large-area-imaging capability can map entire organs, tumors, or whole biopsy, allowing the study of spatial heterogeneity, metabolic gradients, the tumor–stroma line, and localized molecular microdomains [15]. MSI has broad sample compatibility. Techniques such as MALDI-MSI, which can analyze both fresh-frozen and FFPE tissues, retrospective analyses of archived clinical samples become possible for translational research [14].

MSI enables spatial proteomics and biomarker discovery. On-tissue enzymatic digestion (e.g. trypsinization) combined with MS/MS or LC–MS/MS makes peptide identification directly on tissue. Providing spatially resolved protein distribution maps, supporting biomarker discovery and spatial proteomics workflows. It also has flexible spatial and mass resolution. Traditional MSI offers micrometer-scale resolution, while advanced ionization methods like SIMS has submicrometer to nanometer resolution at the cost of molecular range and sensitivity [16].

#### Laser capture microdissection coupled to LC–MS

LCM-MS (laser capture microdissection-mass spectrometry ) combines LCM with liquid chromatography-MS (LC-MS), this creates a very efficient method for spatial proteomics. LCM-MS makes selective sampling possible for tissue microregions that have been accurately identified based on cell clusters or single cells and have undergone thorough proteome-wide analysis [17].

LCM-MS has high spatial precision. LCM can visually identify anatomical or morphological regions. The targeted cells are dissected precisely while maintaining their spatial patterns [18]. There is also deep proteome coverage from tiny samples, the microdissected regions get analyzed by LC-MS, performing simultaneous identification and quantification of hundreds to thousands of proteins [17].

LCM has made major sensitivity improvements with nanoPOTS (nanodroplet processing in one pot for trace samples). This drastically reduces processing volume and sample loss, performing proteomic profiling of tiny tissue fragments, approximately 1000 protein groups from only 10–18 cells. LCM-MS has broad sample compatibility, working efficiently with fresh-frozen and FFPE tissues, supporting both prospective and retrospective clinical studies. It has been successfully applied to human lung tissues (H&E-stained) to differentiate vascular versus alveolar regions and identify ECM proteins. So LCM-MS can be used in translational research, pathology, biomarker discovery, and disease-microenvironment analyses. So with high quantitative accuracy and proteome depth, LCM-MS yields protein identification, quantification, and challenging membrane-associated proteins. In rat brain tissue, LCM-nanoPOTS identified more than 2000 proteins from 200-$\mu $m regions (350 cells, rat hippocampus) with 92% reproducibility and <15% missing data [19]. However, only 18% of transmembrane proteins were detected versus 47% cytosolic, indicating an extraction bias, including membrane, dendritic, and axonal proteins [19].

LCM-MS is adaptable and multimodal, supporting the isolation of well-defined tissue voxels for comparison or mixing. This helps in the construction of spatial proteome maps. Additionally, LCM-MS data are so flexible that they can actually be combined with other spatial omics technologies to make up complete molecular atlases of tissue architecture [20].

#### Multiplexed imaging proteomics

Multiplexed imaging proteomics (MIP) provides scientists with two essential capabilities, which include high multiplexing ability and spatial resolution for performing total protein profiling within unaltered tissue sections. Tissue sections are analyzed using standard imaging mass cytometry (IMC) and multiplexed ion beam imaging (MIBI), as well as DNA-barcode, cyclic fluorescence techniques such as CODEX, and cyclic immunofluorescence methods such as t-CyCIF and IBEX. These techniques can detect between 40 and 100, or more, protein markers in a single tissue section, according to [21]. The system provides higher multiplexing capabilities than traditional immunohistochemistry and simple immunofluorescence methods to perform detailed tissue analysis of complex biological samples.

MIP supports high multiplexing with preserved tissue architecture, imaging proteins within unaltered tissue, maintaining native cellular organization. Mapping of protein expression at cellular and subcellular resolution is possible and essential for understanding cell functions, interactions, and microenvironmental structure [22]. Detailed analysis of complex tissue tissue regions can be studied *in situ*. MIP provides for the detection of rare cell niches, spatially distinct phenotypes, and immune–tumor interactions, which are undetectable in bulk or dissociated-cell assays [23].

There are flexible platforms with complementary strengths for MIP. These are:

- **Metal tag–based methods (IMC, MIBI):** Uses stable isotopic labels to avoid autofluorescence, photobleaching, and spectral overlap. Produces high-quality signals ideal for autofluorescent tissues [22].
- **DNA-barcoded or cyclic fluorescence methods (CODEX, t-CyCIF):** Operates with standard fluorescence microscopes and conventional lab workflows. Increases the accessibility and scalability in basic research settings [24, 25].

#### Single-cell spatial proteomics and low-input MS

MS sensitivity, sample preparation, and microdissection have advanced significantly, which now enable single cells and tiny tissue regions to be analyzed further. This also progresses the understanding of tissue biology, heterogeneity, and disease due to single-cell protein mapping being possible. Low-input MS is designed to detect thousands of proteins from a tiny sample, specifically a single cell.

Marker-free and unbiased proteome profiling in single-cell and low-input MS make proteomics independent of predefined antibodies or transcript-based inference. This makes methods to measure thousands of proteins per cell and examine them without bias, providing a complete view of protein composition in cells. The direct measurement of functional biology, assists MS to provide a more accurate perspective of biological activity. MS quantifies protein abundance, post-translational modifications (PTMs), and proteoforms, revealing regulatory layers undetectable by transcriptomics [26, 27].

SCoPE2 is a major technological milestone; it integrates multiplexing and optimized sample preparation to achieve scalable single-cell proteomics. It can quantify more than 3000 proteins across 1500 human cells, differentiating cell types and proteome-level diversity [28, 29]. Proteomic measurements showed nearly 20 times more protein copies per gene compared to RNA counts, which supports higher precision and signal quality.

#### Subcellular spatial proteomics

When MS techniques are used for the identification of protein locations in cell organelles, it is called subcellular spatial proteomics (SSP) and it makes use of biochemical separation, quantitative proteomics, and statistical modeling.

LOPIT (localization of organelle proteins by isotope tagging) uses partial membrane separation through density and gradient centrifugation, isotope labeling, and MS. The co-fractionation of unknown proteins with known organelle markers assists in organelle assignment without microscopy or antibodies. This provides large-scale mapping of subcellular proteomes. hyperLOPIT which upgrades LOPIT through improved clarity, efficiency, and proteome coverage, can map diverse protein classes (membrane, soluble, and complex-associated) across many organelles [30]. It uses isobaric tag multiplexing, which reduces technical variations and missing data [31, 32].

SSP is dependent on statistical and computational principles. Proteins are assigned to compartments based on similarity to organelle markers after fractionation and MS quantification, alongside multivariate data analysis [31, 33]. This improves precision in identifying protein localization, complexes, and signaling pathways [34]. SSP can detect dynamic protein movement, tracking organelle changes during stress, drug treatment, infection, and disease. These changes are hidden from bulk proteomics or transcriptomics. This is why a systems-level understanding of cell organization helps to reveal how proteins distribute across organelles, move between compartments, form complexes, and define metabolic and signaling modules. Making discovery of new organelle relationships and regulatory networks possible, also supports systems biology and cell behavior research [32].

#### Proximity labeling proteomics

Proximity labeling (PL) proteomics uses modified enzymes, like biotin ligases or peroxidases, joined to a “bait” protein or aimed at a subcellular location, to covalently mark nearby proteins in living cells. Following biotin tagging, these neighboring proteins are concentrated and recognized *via* MS [35]. Scientists established PL as an *in vivo* method to detect proteins in their unaltered cellular environment because it avoids the need for unnatural cell disruption or sample separation.

A key benefit of PL is its capacity to identify temporary, fragile, or shifting interactions and spatial nearness that often avoid more conventional techniques like co-immunoprecipitation or biochemical fractionation [36]. The PL technique functions best to study protein interactions, which exist close to membrane receptors and organelle contact sites and signaling complexes and other small cellular areas because it detects weak and short-lived and rapidly changing interactions [37].

Technological advancements in PL have significantly enhanced its speed and temporal precision. For example, BioID required several hours to biotinylate nearby proteins, in comparison to modified enzymes like TurboID, which can biotinylate nearby proteins within minutes of adding biotin can be considered a significant improvement [37]. PL is reliable for examining cellular processes, signaling events, and active rearrangements due to its precise tracking of local proteomes over time [38]. This is possible because of its rapid labeling approach. PL also has an enzyme tag functionality, which makes assessing proteomes in distinct cellular compartments and subcellular biological contexts possible. It is also possible to obtain nanoscale resolution, allowing protein interactions to be accurately mapped but local environmental information and biological activities must be preserved [39]. This also means we can further understand cellular components by identifying neighboring proteins, temporary interactions, and native cellular structures at high time and spatial resolutions. This makes PL essential for spatial proteomics.

## Computational methods in spatial proteomics

**Fig. 4.**
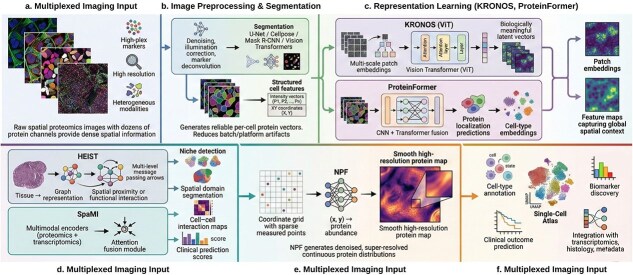
AI/ML in the spatial proteomics.From image preprocessing and segmentation to large-scale self-supervised representation learning (KRONOS, ProteinFormer), spatial graph modeling (HEIST, SpaMI), and neural-field-based continuous reconstruction (NPF). These approaches benefit spatial proteomics through structured single-cell atlases, enabling multimodal integration, biomarker discovery, and clinical prediction.

Spatial proteomics can generate highly multiplexed and high-resolution images that are rich in information and technically heterogeneous. Artificial intelligence and machine learning (AI/ML) methods play important roles in all stages of experimentation and discovery, such as image pre-processing and segmentation, quantitative single-cell feature extraction and normalization, representation learning and cell-type annotation, spatial and graph-based modeling of cellular neighborhoods, and integration with other modalities for biomarker discovery and clinical prediction. These AI/ML approaches together can convert imaging stacks into structured single-cell atlases, which can be used for comparison across platforms, cohorts, and disease states [40]. Figure 4 summarizes how AI/ML models operate within spatial proteomics.

Segmentation performance varies substantially, Cellpose (70K training cells) achieves 0.87 IoU on packed tissues but degrades to 0.61 in high-density regions (>500 cells/mm$^2$); Mesmer (dual nuclear/membrane) reaches 0.79 IoU but requires quality membrane staining; StarDist (star-convex optimization) achieves 0.91 IoU on separated cells but over-segments elongated types (1.8$\times $ false cells versus cellpose). All methods show 15%–25% error in dense microenvironments [41]. These produces reliable cell intensity vectors and spatial coordinates with reduced batch and platform artifacts, improving biological reliability and downstream ML analysis.

KRONOS is a vision transformer pretrained on 26.7M patches from 1024 datasets (47 tissues, 438 markers), achieved 87.3% cell-type accuracy and 0.89 Dice for spatial domains on held-out data. However, performance degraded on underrepresented tissues: 61.2% accuracy for rare neuroendocrine tumors (*n* = 12) and 54.7% for adipose (*n* = 8). The hierarchical ViT architecture (16 $\times \ $16 to 256 $\times \ $256 patches) enables multi-scale extraction but struggles with novel tissue types [42]. This allows it to support cell phenotyping, region classification and patient stratification with strong cross-platform generalization. Similarly another model ProteinFormer combines CNN backbones with global transformers to predict subcellular protein localization [43]. It outperforms pure CNNs, especially with limited data, by integrating long-range spatial organization.

Graph neural networks (GNNs) can represent cells as nodes with protein features and edges encode spatial proximity. GNNs can identify surrounding contexts such as immunosuppressive niches, tertiary lymphoid structures, and invasive fronts; extracting invisible intercellular signals [44]. HEIST models tissues as hierarchical graphs with cells as nodes (spatial edges within 50 $\mu $m) and gene network edges. Pretrained on 22.3M cells from 847 spatial transcriptomics and 124 proteomics datasets using spatial contrastive learning and masked reconstruction, HEIST improved cell-type accuracy by 12.4 points (91.2% versus 78.8%) and reduced imputation error 23% (MAE 0.15 versus 0.19). Critical limitation assumes spatial proximity implies interaction, failing when dissociation introduces artifacts or signaling operates >100 $\mu $m [45]. Making it better in cell-type annotation, clinical prediction, and gene imputation. SpaMI (spatial multi-omics integration) integrates spatial proteomics-transcriptomics *via* dual-encoder GCNs with cross-modal attention, pretrained on 412 matched sections. SpaMI achieved 0.82 protein-RNA correlation (versus 0.71 for Seurat) and identified 89% of histology-validated domains. However, forced alignment masks true discordance SpaMI artificially imposed transcriptomic patterns onto uniformly distributed stable proteins in 14% of cases, obscuring post-transcriptional regulation and protein stability effects [46]. This improves spatial domain detection and data denoising, outperforming earlier multimodal methods.

Joint modeling of spatial proteomics with transcriptomics, histopathology, and metadata can enhance cell-type annotation and biomarker discovery for precision medicine. Public datasets, interoperable toolboxes and their ML pipelines are rapidly evolving essential reproducible benchmarks and cross-platform transfer learning methods [47].

Additionally, neural proteomics fields (NPF) trains a continuous neural function mapping 2D coordinates to protein expression [48]. This reconstructs high-resolution spatial protein maps between measured points, capturing fine-scale gradients. It uses tissue morphology and spatial structure to denoise sparse data and enhance localization accuracy.

### Summary

The developed techniques showcase the primary benefits of spatial proteomics by maintaining biological context while locating protein characteristics. The different approaches generate unique insights about tissue structure and molecular interactions which together build an entire spatial proteome map. Table 1 compares the spatial proteomics techniques detailing their strengths and limitations. Table 2 summarizes the various roles of AI/ML in spatial proteomics. Table 3 details the modeling choices and empirical consequences of methods spatial proteomics.

**Table 1:** Comparison of spatial proteomics techniques, explicitly distinguishing directly measured versus biologically inferred quantities

Technique	Directly measured object	Spatial resolution	Proteome depth	Throughput	Key strengths	Main limitations & inference uncertainty
MSI (MALDI, DESI, SIMS)	Peptide ions (*m*/*z*) at spatial coordinates	10–100 $\mu $m (MALDI); <1 $\mu $m (SIMS)	100–1000 peptides per pixel	Medium (hours per slide)	Label-free; intact tissue; broad molecular coverage; direct peptide detection	Protein identity inferred from peptide mapping (one-to-many ambiguity); abundance inferred from summed intensities; ion suppression; incomplete digestion [16]
LCM-MS	Peptides from microdissected regions	10–100 $\mu $m (region-specific)	1000–3000 proteins per region	Low (15+ hrs per 0.1 mm$^{3}$)	Deep regional proteome coverage; precise ROI selection; LC-MS sensitivity	No direct spatial continuity (region-averaged); protein inference from peptides; labor-intensive serial processing [18]
Multiplex Imaging (IMC, MIBI, CODEX)	Antibody binding intensity (metal isotope or fluorescence) at pixels	1 $\mu $m (single-cell to subcellular)	40–100 markers (antibody-dependent)	Medium-High (slide-level imaging)	Single-cell resolution; preserved morphology; spatial neighborhood analysis	Protein abundance inferred from antibody binding; cross-reactivity (false positives); epitope masking (false negatives); non-linear binding at high concentration [23]
Single-cell MS (SCoPE2)	Detectable subset of cellular peptides	Single-cell ($\sim $10 $\mu $m)	1000–3000 proteins per cell	Low-Medium (100s of cells per run)	Marker-free; unbiased proteome; captures heterogeneity; PTMs detectable	Only 30%–50% of proteome directly observed; missing data non-random; full proteome often imputed statistically [26]
LOPIT/ hyperLOPIT	Protein abundance across subcellular fractions	Organelle-level ($\sim $100 nm, inferred)	5000+ proteins across organelles	Medium (bulk populations)	Quantitative organelle assignment *via* co-fractionation profiles	Organelle localization computationally inferred (no imaging); bulk averaging removes tissue context; requires statistical classification models [30]
Proximity Labeling (BioID, TurboID, APEX)	Biotinylated proteins enriched and identified by MS	Nanoscale proximity (10–50 nm radius, inferred)	100–500 labeled proteins	Medium (requires transfection)	Captures transient neighbors; live-cell compatible; temporal control	Spatial proximity inferred from labeling radius; proximity $\neq $ interaction; promiscuous labeling of abundant proteins; requires genetic manipulation [37]

**Table 2:** Roles of artificial intelligence and machine learning (AI/ML) in spatial proteomics

Area	Role of AI & ML
Image analysis	Segmentation, denoising, quantification
Cell typing	Automated classification
Spatial analysis	Tissue architecture and pattern discovery
Microenvironment	Cell–cell interaction modeling
Clinical modeling	Diagnosis, prognosis, and therapy response
Multi-omics	Cross-modal data integration
Foundation models	Transfer learning across datasets
Automation	Scalable high-throughput analysis

**Table 3:** Key modeling choices and empirical consequences in spatial proteomics

Method	Choice	Consequences	Recommendations
**Segmentation loss functions**			
BCE	Smooth boundaries	18%–24% more merges in dense regions; 8% splits (low SNR)	Use for noisy images
Dice	Sharp boundaries	14% splits in noise; fewer merges	Use for high-quality images
$\alpha $ BCE+(1-$\alpha $)Dice	Tunable	$\alpha $ =0.3 (dense) to 0.7 (sparse); fixed $\alpha $ $\rightarrow $ 10%–15% error	Optimize $\alpha $ per tissue; report merge/split rates
**Foundation model pretraining**			
Contrastive	Co-expression focus	87.3% cell-type accuracy; fails in novel spatial contexts	Use for cell annotation
MAE	Spatial structure focus	15%–20% lower cell accuracy; 22% better domains (Dice 0.89)	Use for domain discovery
Hybrid	Sequential training	Captures both; 2–3$\times $ compute	Use when both critical
**Graph edge definitions**			
KNN (*k* = 5–10)	Density-adaptive	23% asymmetry; false gradients in 18% tissues	Test *k*$\pm $2 sensitivity
Radius ($\epsilon $)	Symmetric	$\epsilon $ = 50: 8.2 (sparse) versus 27.4 (dense) neighbors; 34% over-smoothing	Test $\epsilon $$\pm $20%
Delaunay	Geometric	Fails with segmentation errors (15% spurious edges)	Verify segmentation first
**Neural field smoothness ($\lambda $)**			
Low (<0.001)	Minimal smoothing	Spurious 5–10 $\mu $m hotspots	Avoid unless dense data
High (>0.1)	Excessive smoothing	68% boundaries blurred (5 $\rightarrow $ 25–40 $\mu $m)	Avoid with sharp features
Optimal (0.005–0.02)	Tissue-specific	Lung: 0.015 (16.3% MAPE); Brain: 0.008 (14.1%)	Cross-validate; verify microscopy

## The bad: limitations and trade-offs

**Fig. 5.**
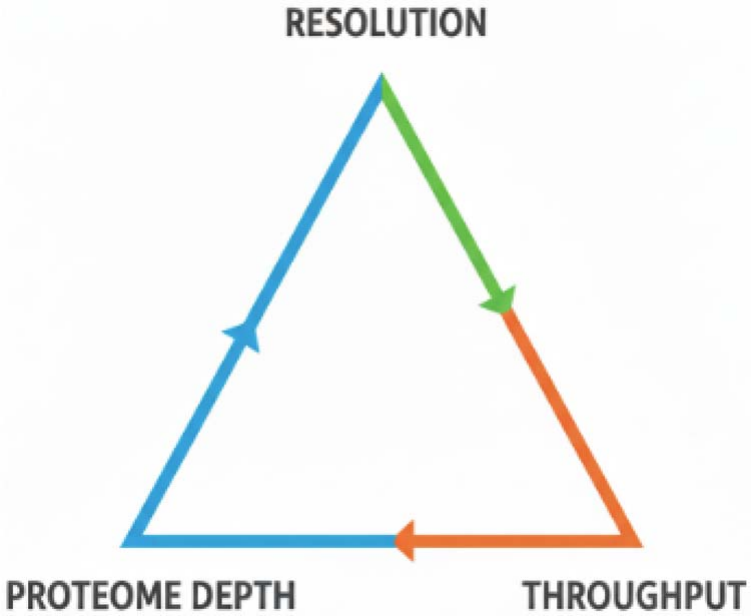
Conceptual illustration of the fundamental trade-offs in spatial proteomics. Higher spatial resolution, deeper proteome coverage, and higher throughput cannot be simultaneously maximized.

While spatial proteomics is powerful, it also faces substantial challenges that limit its current performance, usability, and interpretability. The trade-offs between resolution, proteome depth and throughput as illustrated in Fig. 5

### Sensitivity and input limitations

MS-based spatial and single-cell proteomics face fundamental difficulties, due to extremely limited sample quantities. Spatial voxels, subcellular regions, and single cells provide very small volumes with low analyte concentrations, making peptide separation and enrichment difficult for MS detection [49]. Modern attomole-sensitivity instruments face difficulties as well; only a limited number of proteins from restricted sample amounts [50].

Biased proteome coverage, low input leads to preferential detection of abundant housekeeping proteins. While the low-copy regulatory proteins that control signaling, cell fate, and disease are mostly undetected [51]. This introduces an unintentional selection bias that prevents full system-wide proteome analysis at high spatial resolution [52].

There is also high randomness and missing data, when a protein is represented by only a few peptide ions in a single cell, the quantification becomes noisy, unstable, and poorly reproducible [53]. Taken together with potential losses during sample preparation, e.g. peptide adsorption to surfaces, incomplete digestion or transfer inefficiencies; these limitations can significantly reduce proteome information even before the MS analysis.

Finally, resolution–throughput–proteome depth trade-off ; smaller pieces of tissue or single cells have even less material, so sensitivity and proteome depth decrease further unless one compensates for it by longer instrument time, signal-boosting strategies, or other specialized workflows each of which has trade-offs in terms of throughput, quantification accuracy, or spatial fidelity [54]. Consequently, there is still a fundamental throughput resolution coverage trade-off in spatial proteomics that limits the biological questions that can be addressed reliably.

### Artifacts and quantitative bias in MSI

MSI is an ideal choice for spatial proteomics, but it has inherent technological limitations that limit its quantitative accuracy. Matrix effects and ion suppression reduces quantification accuracy. The local chemical composition strongly influences ionization efficiency, causing the same peptide to generate different signal intensities in different cellular neighborhoods [55]. Even with normalization approaches, these biases cannot be fully resolved, leaving spatial maps vulnerable to errors.

Sample preparation can introduce spatial artifacts. Uneven matrix deposition, tissue thickness variations, and surface topology can create artificial low-signal regions unrelated to protein abundance [56]. These factors generate multi-level batch effects, which reduces reproducibility and complicates cross-laboratory comparisons. A reported radial gradient of PD-L1 in breast cancer MSI was identified as a sample preparation artifact. Re-analysis showed that the pattern correlated perfectly with uneven matrix deposition and trypsin coverage rather than biology, emphasizing the need for mandatory matrix uniformity controls [57].

Inconsistencies in on-tissue digestion worsens variability, the enzymatic digestion efficiency depends on enzyme distribution, local hydration, matrix interactions, and microenvironmental chemistry. Variations in peptide production or delocalization during digestion can misrepresent spatial patterns and intensity from real protein abundance [58, 59]. The lack of peptide separation increases ion suppression and hampers identification. So unlike LC–MS/MS, MSI lacks chromatographic separation, making low-abundance peptides difficult to detect due to competitive ionization [60]. On-tissue MS/MS often produces low-quality spectra, leading to ambiguous peptide identification and biased proteome coverage toward abundant proteins.

Most times, matrix effects, ion suppression, uneven sample preparation, variable digestion, and lack of peptide separation are the root causes of artifacts that make MSI-based spatial proteomics prone to generating spatial patterns that can be misleading, biased quantitation, and low reproducibility [2]. Extreme caution should be exercised when interpreting signal intensities, and it should always be remembered that the differences observed may be the result of technical artifacts rather than true biological variation. Although MSI is still an option to be considered, these limitations are a big bottleneck for accurate, high-coverage, and quantitative spatial proteomics [57].

### Throughput and scalability challenges

While spatial proteomics holds the promise of mapping protein expression in tissue with high resolution, the current workflows still have significant limitations in throughput and scalability, as they tend to severely restrict how much tissue can be processed, how many samples can be analyzed, or how finely the tissue can be resolved.

MS-based spatial workflows suffer from low throughput due to slow microdissection. LCM preserves localization data at high precision, even down to single cells [61]. However, it is very slow and labor-intensive. It needs manual region selection, laser cutting, and capture. A study once employed LCM-MS on human lung tissue required 15 h of microdissection to obtain just 0.1 mm$^3$ of tissue per patient [18]. This proves that processing large areas or multi-patients is unrealistic.

LC-MS processing is a serial and low-throughput bottleneck. LCM generates limited samples, which standard bottom-up LC-MS workflows struggle to process due to high losses, variability, and low reproducibility [62]. Although automated or online digestion methods improve handling, samples are still analyzed sequentially rather than in parallel, setting an upper limit on throughput. As a result, most spatial-proteomics studies have mapped only a few regions per tissue [17].

Antibody-based multiplexed imaging also faces scalability problems. Techniques such as CODEX, IMC, and cyclic immunofluorescence relies on staining, imaging, and fluorophore removal [63]. These are time-consuming and can degrade tissue morphology and antigenicity, requiring continuous improvements to reduce throughput. Higher multiplexing increases data quality but prolongs acquisition, making full-slide or cohort-scale imaging impractical.

So without the significant improvements in automation, parallel processing, and sample handling efficiency becoming commonplace, spatial proteomics will still be constrained to small-scale or highly focused studies instead of large-scale, high-throughput applications.

### Antibody dependence and specificity issues

First off, a lot of imaging-based spatial proteomics, which are dependent on the immune system, are limited in principle by how many, the quality, and the specificity of the antibodies available. Antibodies vary widely in performance; some are highly specific, while others produce weak signals. Antibodies verified by western blot or flow cytometry may not be stable in tissue imaging, leading to false values and poor reproducibility [64]. A single poorly performing antibody can compromise an entire experiment.

Multiplexed cyclic-fluorescence methods can deteriorate data quality, techniques requiring repeated cycles of staining, imaging, and signal removal can damage tissue, degrade epitopes, or alter antigen accessibility [65]. Studies show that approximately 15%–20% of antibodies show increased signal over cycles, while a similar fraction decreases and produces inconsistent intensities, making quantitative comparisons difficult [66]. Spatial patterns may remain stable, but intensity drift introduces marker-specific bias.

Methods like IMC or CODEX require attaching metals or DNA-barcodes to antibodies. A combination can reduce affinity or specificity, requiring re-validation [67]. Barcode-based methods lack signal amplification and struggle to detect low-expression proteins, reducing sensitivity for weak targets [67]. Each antibody must be validated for tissue compatibility, cycle stability, and cross-reactivity. This restricts panel size and bias studies towards well-characterized proteins with reliable antibodies, and limits exploration of novel or low-abundance targets.

Due to these issues that are antibody-specific and workflow-related, sensitivities of imaging-based spatial proteomics could be inconsistent, reproduction of results is extremely difficult, lowly expressed proteins are poorly detected, multiplexing is limited, thus these problems negate the promise of complete spatial proteome mapping. Hence, antibody dependence and specificity are still some of the biggest technical bottlenecks in current spatial proteomics.

### Data complexity and missing values

Spatial proteomic datasets quantify hundreds to thousands of proteins across numerous spatial locations, which creates high-dimensional and structured data. Advanced statistical and ML methods, such as non-parametric Bayesian models are needed to capture spatial correlations and protein interactions efficiently [33]. But these models are very computationally intensive, which reduces their adoption and prevents full exploitation of complex datasets.

MS-based spatial and single-cell proteomics often fail to detect low-abundance proteins or small-input samples, this results in 10%–50% missing data in label-free workflows [68]. Missingness can be MNAR (missing not at random) or MAR (missing at random) [69]. High missing data reduces reliability, reproducibility, and interpretability of quantitative results, so imputation is needed. Common imputation strategies include single-value replacement, k-nearest neighbors (kNN), or global-similarity methods. Improper imputation can introduce bias and false patterns, which can underestimate uncertainty especially when missing data is evident [70]. MNAR scenarios are very problematic because methods assuming random missingness can produce incorrect results.

Besides, in cases where MNAR, the methods that work under the assumption of random missingness may deliver incorrect results or be ineffective [71]. A few more advanced solutions try to tackle this problem, e.g. new statistical frameworks that assign less weight to less confidently imputed values or that identify missingness during inference instead of simply filling in the gaps. However, such techniques are still in the testing phase, and the risk is still there that the spatial proteomic discoveries that are mistakenly thought to be biological phenomena may actually be the result of the imputation artifacts, until they are extensively used for interpretation and discovery of real biological patterns [71].

### Integration challenges with other omics

Spatial proteomics often produces imaging-based or MS-derived continuous or sparse protein-abundance data, whereas transcriptomics generates discrete count matrices per gene per cell; integrating these fundamentally different data structures requires mapping imaging grids or microdissected regions to sequencing spots or single-cell transcript counts [3]. While spatial proteomics can achieve subcellular resolution for a limited protein panel, spatial transcriptomics captures the full transcriptome at lower spatial precision, creating resolution mismatches in which protein measurements originate from small regions whereas transcriptomic spots average broader neighborhoods, complicating direct alignment [72, 73]. Emerging multimodal frameworks and graph-based integration strategies attempt to address these challenges through resolution-aware modeling and contrastive pretraining, yet they also highlight the statistical and computational complexity inherent in cross-modal alignment [74, 75]. Compounding this difficulty, no community-standard pipelines or metadata frameworks currently exist for integrating spatial proteomics with other omics, and differences in data formats, noise distributions, antibody panel bias, sample preparation protocols, and platform-specific artifacts further undermine reproducibility [72]. As a result, without careful registration, probabilistic alignment, and standardized reporting practices, multimodal spatial integration risks producing ambiguous or irreproducible conclusions, underscoring the urgent need for harmonized standards, uncertainty-aware modeling, and adaptable computational infrastructure capable of integrating heterogeneous molecular modalities.

Integrating spatial proteomics with other omics also faces five technical problems that demand tailored analytical solutions. First, spatial registration across serial sections introduces 5–15 $\mu $m alignment errors that misassign 20%–35% of cells; morphology-guided tools like STalign or feature-based methods like PASTE reduce error to 3–4 $\mu $m, but residual uncertainty must be quantified and propagated [20, 76]. Second, resolution mismatch proteomics at 1–10 $\mu $m versus transcriptomics at 10–100 $\mu $m inflates correlations when naively integrated. Recommended approaches include decomposition to cell types (Cell2location) or resolution-aware probabilistic models rather than direct correlation or aggressive imputation [77]. Third, antibody panel bias toward functional markers creates misleading correlations when integrated with unbiased transcriptomes; stratified analysis by protein class or propensity weighting corrects this asymmetry [21].

The remaining challenges concern data characteristics and interpretation. Fourth, mismatched noise distributions, negative binomial RNA counts versus log-normal protein intensities increase false positives under Gaussian assumptions. Variance stabilization and distribution-aware methods are essential [78]. Fifth, RNA-protein discordance (median *r* = 0.3–0.5) reflects both biology and artifacts; protein half-life differences explain much variance, while antibody cross-reactivity contributes technical noise. Rather than forcing agreement, research should stratify by protein class, account for turnover rates, validate antibodies, and treat discordance as a regulatory signal [25]. Collectively, these frameworks transform integration from a technical obstacle into a tool for discovering post-transcriptional regulation and multi-scale tissue organization [79].

Table 4 serves as a methodological guide for integrating spatial proteomics and other omics data. It outlines common technical pitfalls in multi-omic spatial analysis and provides best-practice solutions for handling data generated from different modalities.

**Table 4:** Challenges and recommendations for integrating spatial proteomics and transcriptomics data

Challenge	What NOT to Do	Recommended approach
Registration	Ignore alignment error; assume perfect registration	Use morphology-guided registration (STalign); report error; exclude misaligned regions
Resolution mismatch	Directly correlate 5 $\mu $m proteomics with 55 $\mu $m transcriptomics	Integrate at intermediate scale (10–30 $\mu $m) or decompose to cell types (Cell2location)
Panel bias	Train models on biased 40-protein panel; apply to full transcriptome	Stratified analysis by protein class; propensity weighting; validate on mass-spec
Noise distributions	Combine raw RNA counts with raw protein intensities	Variance-stabilize RNA; log-transform protein; use distribution-aware methods (MultiMAP)
RNA-protein discordance	Interpret low correlation as data quality problem; force agreement imputation	Stratify by protein class; account for half-life; validate antibodies; leverage as regulatory signal

## The ugly: critical pitfalls, artifacts, and risks of misinterpretation

There are also “ugly” pitfalls in spatial proteomics that can lead to inaccurate biological results if not managed properly. These are frequently ignored but are crucial for robust spatial proteomics.

### False spatial gradients and artifact-driven patterns

One of the greatest concerns is that technical problems, instead of real biology, might make it look like spatial patterns exist. To illustrate, with MSI methods, a non-uniform sample preparation, uneven matrix deposition, inconsistent ionization, or variable digestion efficiency can result in an area being a “gradient” of protein abundance across a tissue section [56, 60]. This gradient might be wrongly interpreted as a biological zonation or a real cellular spatial gradient when, in fact, it is just an artifact of the technique. Sample preparation can indeed very much affect detection sensitivity and if spatial controls are not very strict, the resulting artifacts may be taken for localization differences [2, 55].

Such false gradients can be highly deceptive since they might appear plausible; for instance, tissues are usually structured in a heterogeneous way. However, if technical variations coincide with those structural boundaries, e.g. in some areas there is differential ionization, which might wrongly conclude that there is a biologically significant protein localization when in fact it is just a technical bias [19, 80].

### Mis-segmentation in imaging-based methods

In imaging-based spatial proteomics, the cell segmentation algorithms are very important: they determine which pixels correspond to a specific cell. If the segmentation is not correct, either by excessively dividing or not dividing at all, the distribution of the protein signal to the cells will definitely be unreliable [81]. Such misassignment can cause very serious errors in further studies, like wrong-type cell inferring, signal mixing from adjacent cells, or incorrect calculations of cell–cell interactions and neighborhood relationships.

Correct segmentation is a prerequisite for many downstream conclusions, including cell type identification, spatial organization, and cell–cell interaction networks, thus segmentation errors can completely destroy the biological validity of the data being analyzed. Over-segmentation might falsely increase cell counts or create artificial cell types; under-segmentation might make cellular boundaries disappear and mix up distinctions between cell types. If not carefully validated or manually inspected, such errors may spread throughout the entire spatial analysis [82]. Computational identification of “transitional $\beta $-cells” in pancreatic islets CODEX was revealed to be a result of under-segmentation in high-density regions. Manual curation found that 94% of these cells were actually merged multi-cell doublets where the algorithm failed to resolve boundaries in packed islet cores [83].

### Protein delocalization during sample processing

One more issue that is often overlooked comes from the sample preparation and it is considered one of the main issues. During the processing of the tissue, the washing, antigen retrieval, fixation, staining, or on-tissue digestion may lead to the diffusion or washing away of the proteins from their native locations. This “delocalization” has a negative impact on the actual spatial distribution of the proteins.

An example of one such case is the MS-based spatial proteomics where peptide extraction and digestion can cause leaching or redistribution and in that way smearing the spatial specificity. Harsh retrieval methods used in imaging-based techniques may wash out weakly bound proteins or lead to the redistribution of the proteins across the section. Consequently, a protein that was once confined to a specific subregion or cellular compartment might end up being evenly distributed in the final data, thus leading us to the wrong interpretation of its original, biologically relevant spatial pattern [6]. Apparent nuclear-to-cytoplasmic redistribution of FOXP3 in regulatory T cells was found to be artifactual leakage caused by room-temperature incubation prior to fixation. Rapid fixation ($<2$ min) or cross-linking confirmed exclusively nuclear localization, highlighting the susceptibility of soluble nuclear proteins to extraction during processing [84].

This problem is particularly severe with soluble proteins or those having weak interactions with cellular structures. Even the methods used to fix and preserve tissue architecture do not completely eliminate the risk: for instance, cross-linking can hinder extraction or antigen retrieval might not reverse cross-links thoroughly, thus biasing further which proteins are actually detected [72].

### Overinterpretation without orthogonal validation

One of the main advantages of spatial proteomics is the creation of very impressive maps showing the localization of different proteins. It is quite easy to get carried away, especially for new biological insights or biomarkers, when presented with such attractive heatmaps or tissue overlays, to come up with conclusions that are strong [25]. Nevertheless, it is more than likely that such conclusions will be incorrect or made too early if not supported by rigorous orthogonal validation [85].

If one method is the only source of data, then it is easy to confuse the technical noise, staining artifacts, batch effects, or segmentation errors with random biological variation [86]. At worst, if one dataset is the only source of data, then a novel spatially enriched protein or new cell-cell interaction zone might be declared based on that one dataset, which might not reproduce under different conditions, samples, or laboratories [87].

Consequently, it is imperative that spatial proteomics discoveries be confirmed through independent methods like classical immunohistochemistry, *in situ* hybridization, functional assays, or repeated experiments across different samples or cohorts. If such validation is not performed, there will be a very high chance of misinterpreting artifacts as biologically significant phenomena [88].

### Batch effects masquerading as spatial differences

Due to the complexity of spatial proteomics workflows that involve tissue preparation, imaging or microdissection, digestion or extraction, MS (or multiplex imaging), computational segmentation, and bioinformatic analysis altogether, it is very hard to replicate the same experiment in different labs. It could also be that different protocols, different types of samples, different antibodies, different microdissection strategies, or different computational pipelines lead to totally different results [71, 89].

In fact, it happens that the same tissue type is used and different labs still report different “spatial proteomes.” This inconsistency in results makes it impossible to compare studies, limits meta-analysis, and slows down the overall use of spatial proteomics in biological and clinical research. A major barrier lies in the absence of harmonized sample preparation, data processing, and analysis pipelines [69, 72].

The problem of the high dimensionality and complexity of spatial proteomics data also contributes to the issue of ensuring reproducible computational analyses being difficult, especially when the labs are using different software, segmentation tools, or thresholds [90].

Table 5 summarizes the root causes and the amplifying factors of each pitfall and also their mitigations.

**Table 5:** Critical pitfalls in spatial proteomics and mitigation strategies

Pitfall	Root cause	Amplifying factors	Mitigation
**False spatial gradients [19, 55, 56]**	Uneven sample prep creates position-dependent artifacts mimicking biology	Fitting smooth functions without technical covariates; ignoring spatial autocorrelation	Model position or batch explicitly; validate with IHC/IF; use uniform controls
**Batch effects as biology [56, 69, 71]**	Multi-step workflows introduce systematic variation confounded with biology	Single-batch training; linear correction removing true biology	Randomize design; batch-aware normalization; validate across cohorts
**Segmentation errors [81–83]**	Cell merging or splitting misassigns signals, creating false cell types	Single algorithm without QC; declaring novel types without validation	Ensemble segmentation; manual validation; filter low-confidence boundaries
**Missing data bias [69–71]**	Low-abundance proteins systematically missed (MNAR); imputation creates false patterns	Simple imputation (zeros, mean) assuming MAR; ignoring uncertainty	MNAR-aware imputation; report missingness; prioritize proteins <20% missing
**Protein delocalization [17, 18, 84]**	Soluble proteins diffuse during processing, blurring native compartments	Harsh prep; interpreting artifacts as redistribution	Rapid fixation (<2 min); cross-link first; validate with IF/IHC
**Spatial autocorrelation [7, 25, 86]**	Neighboring cells non-independent; standard tests inflate significance	Treating cells as independent; no spatial FDR correction	Spatially-aware models (GEE, GP); calculate Moran’s I; adjust effective $N$

### AI/ML failure modes in spatial proteomics

AI and ML have accelerated spatial proteomics analysis but introduce specific failure modes that generate false biological insights if not carefully managed. The high-dimensional, sparse, batch-affected nature of spatial proteomics data creates acute risks: overfitting on small datasets (10–50 tissue sections with millions of model parameters), domain shift across platforms (KRONOS accuracy drops from 89% to 54% on novel antibody panels), hallucinated spatial structures from generative models (NPF interpolation creating false transition zones at genuine boundaries), and overconfident predictions despite class imbalance and noisy labels. Foundation models like KRONOS, HEIST, and SpaMI each exhibit method-specific vulnerabilities tied to their architectural choices—vision transformers struggle with unseen markers, graph models assume spatial proximity implies biological interaction, and multimodal aligners can force agreement where modalities genuinely disagree. Mitigation requires systematic validation practices: report training/validation/test performance separately with confidence intervals, test on out-of-distribution data (different tissues, platforms, disease states), provide uncertainty estimates for all predictions, perform ablation studies, use negative controls (shuffled coordinates, synthetic ground truth), and distinguish measured versus model-generated content in all figures. Calibration techniques (temperature scaling, Platt scaling) align confidence scores with true accuracy. Most critically, validate all computationally-identified patterns with orthogonal methods (microscopy, independent cohorts, alternative algorithms) before biological interpretation. Table 6 summarizes failure modes, mechanisms, and mitigation strategies for major AI/ML methods in spatial proteomics.

**Table 6:** AI/ML failure modes, mechanisms, and mitigation strategies in spatial proteomics

Failure mode	Mechanism & evidence	Method-specific risks	Mitigation strategies
**Overfitting on small datasets**	10–50 tissue sections with millions of parameters; models memorize noise. GCN study: 98% training $\rightarrow $ 62% test accuracy	KRONOS/HEIST: Fine-tuning on small datasets overfits to technical artifacts (staining, thickness). Spurious co-expression from batch effects	Early stopping; dropout; L1/L2 regularization; report train/val/test separately; independent cohorts; ablation studies
**Domain shift across platforms**	Same tissue, different platforms (IMC versus CODEX) $\rightarrow $ different features. Models fail catastrophically on new domains	KRONOS: 89% $\rightarrow $ 54% accuracy on novel antibody panels. Foundation models inherit training bias (tumor-heavy data misclassify normal tissue)	Test on multiple platforms/tissues; report stratified metrics; domain adaptation; retrain/fine-tune for new applications
**Hallucinated spatial structures**	Generative models (NPF) create plausible but fictitious patterns over-smoothing, prior-driven reconstruction. No uncertainty estimates	NPF: Blurs sharp boundaries into false transitions. GNN imputation: imposes training priors (immune–stroma patterns) onto absent data	Report uncertainty/confidence maps; validate with orthogonal imaging; ablation studies; distinguish measured versus model-generated
**Model over-confidence**	High confidence on incorrect predictions; exacerbated by class imbalance, noisy labels. Softmax $\neq $ true probability (90% conf. = 60% acc.)	HEIST: 95% confidence on ambiguous inputs. Rare cell classifiers default to majority class with high confidence	Calibration (temperature/Platt scaling); dropout sampling; Bayesian NNs; report calibration error; flag low-confidence
**KRONOS vision transformer**	Pretrained on diverse imaging; excels at transfer learning	Fails on: (1) absent markers, (2) novel spatial arrangements, (3) extreme class imbalance	Validate on target tissue; check marker overlap; avoid fine-tuning on <100 samples
**HEIST graph transformer**	Cells as nodes with spatial edges; rich neighborhood context	Assumes proximity = interaction; fails when: (1) dissociation artifacts, (2) long-range signaling, (3) segmentation errors	Validate neighborhoods; test edge sensitivity; verify segmentation quality
**SpaMI multimodal**	Aligns proteomics with transcriptomics cross-modal attention	Forces alignment where modalities disagree; propagates errors; overweights dominant modality	Expect RNA–protein discordance; validate with separate modality controls

## Discussion

This review has systematically examined the strengths, limitations, and critical pitfalls of spatial proteomics. The field requires immediate attention to validation standards, reproducibility frameworks, and artifact mitigation strategies to realize its translational potential. From this paper, it can be deduced that there is an enormous need for better methods, analysis, validation, and rigor for spatial proteomics to develop new biological concepts.

Spatial proteomics is a rapidly evolving field, so sensitivity and throughput will continue to improve. The next-generation of MS and ion sources will reduce losses due to higher sampling efficiency and sample preparation. Multiplexed imaging is advancing towards the higher detection of biomolecules through improved barcoding and spectral methods. These advances in imaging are expected to create hybrid methods that combine it with MS, becoming a rising category of methods in spatial proteomics.

The progressive development towards shared protocols and benchmarks is slowly becoming a major characteristic of spatial proteomics, as it slowly emphasizes reproducibility and standard. The Spatial Touchstone project, which was adopted in spatial transcriptomics, demonstrate the advantages of multi-site standard operating procedures (SOP) and open quality-metric tools [91]. So similarly its can be expected that such comparable standards can be developed for spatial proteomics. This is why the processes of locating reference materials, reporting guidelines, and computational benchmarks will become essential for datasets from various labs can be comparable and maintain standards. Stricter validation pipelines through orthogonal assays or genetic controls, have the potential to become the norm in order to prevent excessive claims of novelty [91].

Integration with multi-omics and clinical environment represents the most significant challenge of spatial proteomics. This will then be further combined with data from spatial transcriptomics, metabolomics, and imaging to generate tissue datasets that are comprehensive [92]. These datasets can then be used for clinical purposes such as refined disease subtyping, predicting treatment resistance, guiding drug discovery and many more. To accomplish this workflows need to become automated, and the outcomes should be linked to clinical endpoints.

The field will also be influenced by the most advanced AI models and standards. Models such as KRONOS will directly assist in transfer learning for novel tasks and rare diseases. Generative AI might also open the pathway for in silico experiments. It would also be possible to simulate multiplexed slides or predictions for proteomic patterns from sparse data [93]. Additionally, these models should be developed further for better biological and clinical understanding, attention-based analysis and explainable AI; making the models more stable and accurate in pointing out the proteins or spatial features towards a prediction.

Through continued technological innovation with strong computational and validation frameworks, spatial proteomics can provide tissue biology with novel insights. As multiplexed imaging, single-cell proteomics, and AI-driven analysis combine, it can be expected that spatial proteomics to become crucial in basic research, translational studies, and precision medicine [92].

The future directions of spatial proteomics can be grouped into priorities which are listed below, from this review. Future research focused on these can accelerate spatial proteomics towards greater dependability, accessibility, and biological utility:

- **Standardization and benchmarking** There is a need for protocols and benchmarks that standardizes validation in spatial proteomics. These can be beneficial for sample preparation, spatial resolution, proteome depth, pixel size, and normalization pipelines. Consistent standards will prevent biased comparisons across technologies and increase reproducibility.
- **Integrated multi-modal spatial omics** By combining spatial proteomics data with other data modalities such as spatial transcriptomics, metabolomics, imaging, and computational tissue datasets, will provide deeper insights into spatial biology. The limitations of any single modality can also be overcome and improve integrative analyses.
- **Improved sensitivity and miniaturized workflows** Continuous development in MS, laser-capture micro-dissection, and next-generation ion sources will essentially advance peptide recovery and detection from small regions and single cells.
- **Transparent computational practices** As ML and statistics become more integral for spatial proteomics, transparent reports from models, parameters, and uncertainty are crucial. Necessary steps should be taken to avoid overfitting and misinterpreting high-dimensional spatial patterns.
- **Generative AI and virtual spatial proteomics** Generative AI and virtual systems will be essential in imputing missing protein channels, predicting spatial distribution of proteins and simulation of virtual multiplexed tissues. These will reduce cost and improve biological interpretability.
- **Explainable AI for biological discovery** Explainable AI will be crucial for spatial proteomics to go beyond the confines of black-box prediction. This will improve the trust in spatial proteomics as a clear view of processes related to protein classification, interpretable spatial neighbors and biologically traceable model decisions are obtained.
- **Biological interpretation with caution** Although spatial proteomics data are rich and visually compelling, their interpretation must remain biologically plausible, technically informed, and supported by reliable evidence.

## Conclusion

Spatial proteomics is gaining significant advancements, largely credited to a series of improvements in several domains such as tissue sampling, MS, imaging-based procedures, computational developments, and multimodal integration. All these have developed spatial proteomics to go beyond static and low-resolution images. But the full potential of spatial proteomics cannot be argued if obstacles like sample preparation biases, data missingness, poor resolution, and artifacts resulting from computational processes still impact it.

Many of these issues arise from batch correction and imputation to spatial reconstruction, multimodal fusion, and predictive modeling. These can be mostly solved by ML/AI methods. But ML/AI methods should be integrated into spatial proteomics carefully, while accounting for the need of interpretability, domain shifts, and problematic variables. The rapid development of the field is being led towards large-scale datasets, time-resolved designs, sparse-sampling acceleration, and cellular level details, which give numerous opportunities for the next generation of computational models, including generative frameworks, foundation models, and spatiotemporal predictors.

The combination of cutting-edge experimental processes with strict, interpretable ML/AI will lead spatial proteomics to provide more detailed insights into tissue microenvironments, accurate disease heterogeneity mapping, and implementable precise medicine biomarkers. This is a long process that requires interdisciplinary collaboration, standardization, open data-sharing, and reproducible science. Through a commitment to these principles, spatial proteomics will not only improve current techniques but also transform into a core practice of spatial biology and clinical research.

**Key Points**

- Categorization of the strengths, limitations, and critical pitfalls of spatial proteomics.
- Discussion of major spatial proteomics technologies.
- Identification of crucial experimental and computational challenges.
- Analysis of misleading artifacts.

## Data Availability

None declared.

## Supplementary data

None declared.
